# A Rare Presentation of Polyangiitis Overlapping Syndrome

**DOI:** 10.7759/cureus.36626

**Published:** 2023-03-24

**Authors:** Anita Subramanian, Andrew Hanchosky, Sharmilee Vuyyuru, Kyle Coffey, Therese Massri, Christopher Stewart

**Affiliations:** 1 Internal Medicine, Harnett Health System, Campbell University, Lillington, USA; 2 Medicine, Ross University School of Medicine, Bridgetown, BRB; 3 Internal Medicine, Campbell University School of Osteopathic Medicine, Lillington, USA

**Keywords:** egpa, granulomatosis with polyangiitis (gpa), polyangiitis, cutaneous lesions, oral ulcers, endocarditis, antineutrophil cytoplasmic antibody (anca) associated vasculitis (aav)

## Abstract

This case follows a 38-year-old Caucasian male with no known medical history who presented to the emergency department for syncope. He also endorsed a two-month history of fevers, weight loss, oral ulcers, rashes, joint swelling and arthralgias. After extensive workup, he was given a working diagnosis of granulomatosis with polyangiitis (GPA). Conflicting diagnostic evidence made it increasingly difficult to distinguish between GPA and eosinophilic granulomatosis with polyangiitis. In conclusion, we believe the patient may be better diagnosed with polyangiitis overlapping syndrome.

## Introduction

Small vessel vasculitides are a group of diseases that primarily affect small blood vessels including arterioles, capillaries and venules. The three antineutrophil cytoplasmic antibodies (ANCA)-mediated small vessel vasculitides include granulomatosis with polyangiitis (GPA), eosinophilic granulomatosis with polyangiitis (EGPA) and microscopic polyangiitis (MPA) [1]. The most common organs affected in this cluster of diseases are the kidney and lungs which are further differentiated based on their clinical, laboratory and pathological findings. The most common distinguishable findings for EGPA is the clinical presentation of asthma in 96-100% of patients, while symptoms of the ear, nose or throat are seen in 70-100% of GPA patients [2,3]. While there are commonalities between the clinical presentation of EGPA and GPA, laboratory data helps further discern between them. Positive C-ANCA along with positive PR3 antibodies is found in 80-90% of GPA patients and in 5% of EGPA cases, while positive perinuclear-ANCA or P-ANCA is seen in 40% of EGPA patients and 25% of GPA cases [2,4,5].

The American College of Rheumatology developed diagnostic criteria for both EGPA and GPA. In EGPA there are six characteristic criteria described: Eosinophilia greater than 10%, asthma, neuropathy, lung infiltrates, paranasal sinus abnormalities and tissue biopsy revealing eosinophilic infiltrate [2,6]. Eosinophilia of greater than 10% of the total leukocyte count or greater than 1,500 cells/dL is one of the essential diagnostic criteria for EGPA [2]. The inclusion of four out of the following six criteria carries a sensitivity of 85% and a specificity of 99.7% for diagnosis. The criteria for GPA include abnormal urinary sediment (red cell casts or greater than five red blood cells per high-power field), abnormal findings on chest radiograph (nodules, cavities or fixed infiltrates), oral ulcers or nasal discharge and granulomatous inflammation on biopsy. If at least two of the criteria are found, the diagnosis carries a sensitivity of 88% and a specificity of 92% [7]. In this case report, we will present a unique case of polyangiitis overlap syndrome. Although there are no clear and concise diagnostic criteria to describe the syndrome, it is defined as a systemic vasculitis that cannot be classified into one of the other well-defined vasculitis syndromes. We will explore the clinical presentation, identification and management of this small vessel vasculitis.

## Case presentation

**Figure 1:**
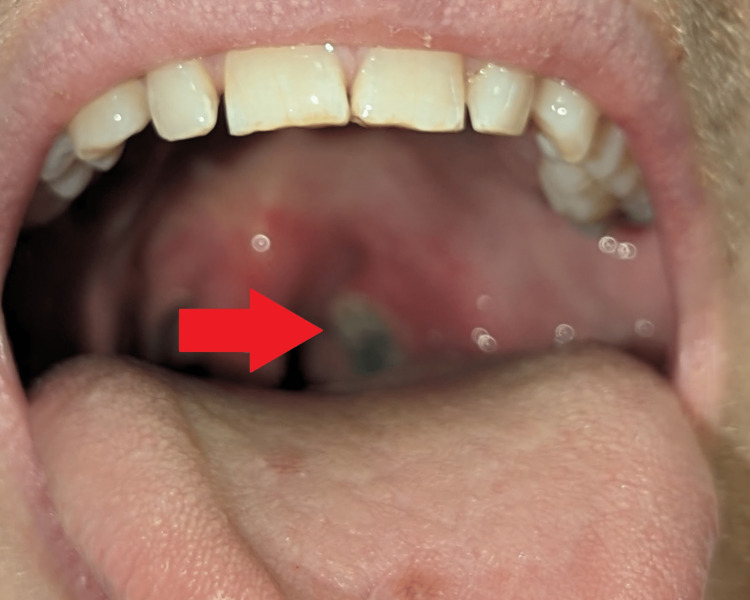
Oral lesions in the patient's mouth

**Figure 2:**
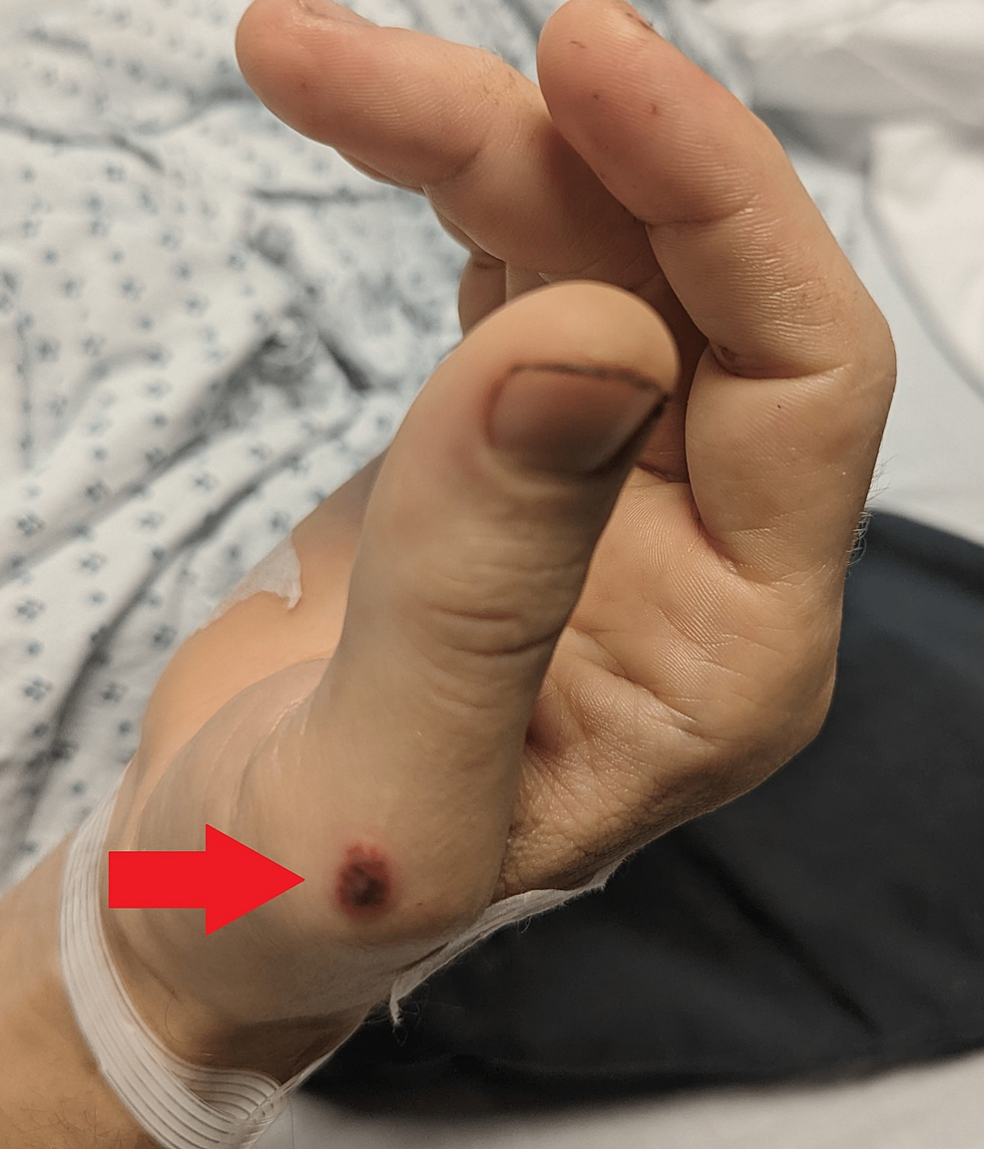
Lesions on patient's hand

The patient is a 38-year-old Caucasian male who initially presented to our emergency department (ED) after multiple syncopal episodes with associated lightheadedness. On further history, the patient reported a two-month history of significant weight loss of greater than 12 pounds, arthralgias, joint swelling, oral ulcers, rashes on the hands and feet, intermittent fevers and multiple syncopal episodes. Lesions on the hand and mouth started a few weeks prior to the presentation to our ED (Figures 1-2). Oral ulcers were painful, approximately 1cm in size, and erythematous with a greyish membrane. Lesions on the hand as depicted below were approximately 1-2cm, papular, erythematous and painless.

The patient was initially on prednisone 40mg daily for these symptoms per his primary care physician and had mild improvement in pain and joint swelling but did not finish the course. Post-treatment the patient presented to an outside hospital ED with complaints of a rash on bilateral hands and feet, easy bruising, painful oral ulcers and erythematous eyes with excessive tearing. The patient was found to be anemic with a hemoglobin of 9, given a dose of dexamethasone and discharged with hematology follow-up.

The patient had no improvement in his symptoms after discharge and presented to our ED one week later for continued multiple syncopal episodes with presyncope, no precipitating factors, varying time between episodes and no other obvious causes such as medications. He did endorse a camping trip prior to symptom onset but denied any tick bites, insect bites or viral illnesses. Following this initial incident, he developed an episode of unilateral atraumatic right shoulder pain that progressed to multiple joints with visible swelling in bilateral ankles and wrists. The patient reported these episodes were more frequent and the pain was worse in the morning, with no change in activity and no alleviating factors.

On arrival, his vitals were significant for tachycardia, positive orthostatic vitals, and a fever of 101.5°F. Labs revealed a leukocytosis of 15.6 with 33% eosinophils and hemoglobin of 9.1. He denied any history of illicit drug use, recent illnesses, malignancies or diagnosis of rheumatologic diseases. The patient was previously in the military and served in Iraq and Afghanistan and current occupation as a home inspector. He did note exposure to dogs and chickens, and also endorsed recent travel history to Maine and Florida. His family history was significant for psoriatic arthritis in his grandfather and hypothyroidism in his mother and brother.

**Figure 3:**
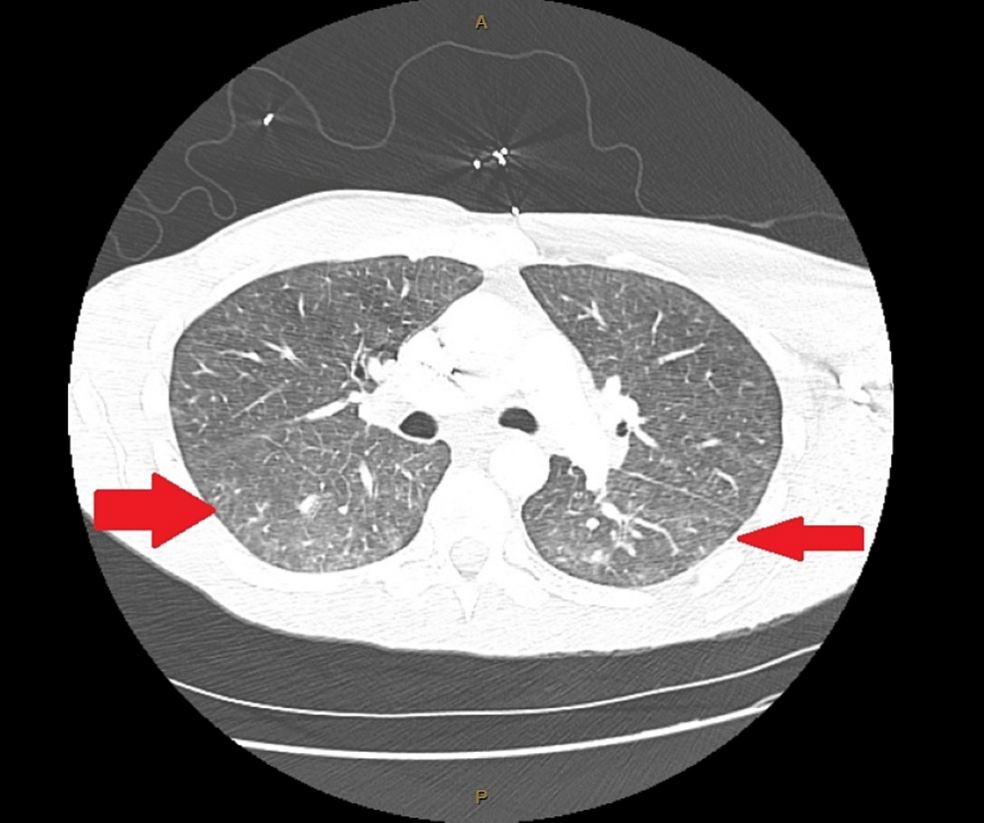
CT chest showing ground-glass opacities and pulmonary nodules CT: computed tomography

On presentation, the patient was noted to have a purpuric rash on bilateral hands, petechial rash on the feet, painful oral ulcers and bilateral conjunctival injection with tearing of the eyes. The patient had a negative rapid plasma reagin (RPR), rapid strep antigen test, IgG Lyme serology, rocky mountain spotted fever IgM, chlamydia/gonorrhea, Human Immunodeficiency Virus (HIV), Anti-nuclear antibody (ANA) panel, hepatitis panel and anti-cyclic citrullinated peptide. He had a significantly elevated ESR of 81, CRP of 47 and an elevated RF of 76. Additionally, the patient was found to have positive CMV IgG and EBV IgG antibodies. Computed tomography (CT) scan of the chest, abdomen and pelvis showed multiple pulmonary nodules measuring up to 8mm and bilateral ground-glass opacities in both lungs (Figure 3). During hospitalization, the patient refused steroid therapy until a final diagnosis was determined, so he was discharged home once symptoms improved with rheumatology follow-up.

The patient returned to the ED two days post-discharge for another syncopal episode with concurrent hypotension and was transferred to a tertiary care center due to limited resources at our community hospital. During his hospital course at the outside facility, the patient had worsening eosinophilia and anemia and declining respiratory status requiring oxygen supplementation. A repeat CT chest/abdomen showed aortic valvular vegetations concerning endocarditis and wedge-shaped renal and splenic infarcts new from previous imaging. MRI/MRA brain demonstrated recent infarcts in bilateral supratentorial and infratentorial compartments compatible with central embolic etiology given cardiac vegetations. Laboratory studies revealed IgE 809, normal complement levels, positive C-ANCA 1:1280 and Anti-proteinase 3 (PR-3) > 100, with a negative anti-myeloperoxidase antibody. Multiple skin biopsies performed showed perivascular infiltration with eosinophils and neutrophils with a small amount of karyorrhectic debris concerning EGPA. With the development of a hyperpigmented macular rash, repeat skin biopsies were performed which showed unremarkable epidermis associated with small vessel vasculitis with eosinophils supporting a differential diagnosis of leukocytoclastic vasculitis and EGPA. The patient was started on Solu-Medrol 40mg IV every 8 hours and received Rituximab induction therapy. Transesophageal echocardiogram revealed moderate to large echogenic masses seen on each of the three aortic leaflets on the aortic surface most consistent with organized vegetations. The patient eventually underwent an open sternotomy with cardiopulmonary bypass mechanical aortic valve replacement and wedge resection of the lung for pathology with some improvement in respiratory symptoms.

The patient had an unremarkable postoperative course and was successfully discharged home for continued recovery. The patient was discharged home on Prednisone taper and warfarin for valve replacement. Bone marrow biopsy demonstrated normocellular marrow (60%) with mild eosinophilia (15%). Aortic pathology showed focal myxoid degeneration, granulation tissue and attached fibrin clots with macrophages, eosinophils and mild acute inflammation and lung resection displayed a constellation of findings including abundant intra-alveolar macrophages with smoker's pigment and eosinophils. This presence of variable degrees of infiltration of vessels by eosinophils overlaps significantly with EPGA. While findings were incredibly difficult to differentiate between EGPA vs GPA, based on serology findings of positive PR3 and a history unremarkable for asthma or allergic rhinitis on discharge he was given a working diagnosis of GPA per the rheumatology team.

## Discussion

Our patient initially presented with a plethora of symptoms including syncopal episodes, weight loss, bilateral lesions on his hands and feet, arthralgia, myalgia, conjunctival erythema, photophobia, oral ulcers and sinusitis. Table 1 below depicts the symptoms typically indicative of GPA vs EGPA. These symptoms were associated with initial findings of normocytic anemia, elevated erythrocyte sedimentation rate, c-reactive protein, rheumatoid factor and significant eosinophilia, a working diagnosis of EGPA was favored with 60% of patients having similar results [8]. However, the absence of asthma and positive C-ANCA and PR-3 results questioned our initial thoughts.

**Table 1:** Patient’s pertinent signs/symptoms associated with EGPA, GPA or both EGPA: eosinophilic granulomatosis with polyangiitis, GPA: granulomatosis with polyangiitis, ESR: erythrocyte sedimentation rate, CRP: C-reactive protein, RF: rheumatoid factor, C-ANCA: c-antineutrophil cytoplasmic antibodies, PR-3: proteinase 3

Sign, Symptom or Finding	Associated with EGPA	Associated with GPA	Common to Both
Syncope			X
Fever			X
Weight Loss			X
Arthralgias			X
Joint Swelling			X
Oral Ulcers		X	
Hand and Foot Rash			X
Conjunctival Injection		X	
Anemia			X
Leukocytosis			X
Eosinophilia	X		
Elevated ESR		X	
Elevated CRP		X	
Elevated RF	X		
Elevated IgE	X		
Positive C-ANCA and PR-3		X	
Bilateral Lung Opacities			X
Pulmonary Nodules		X	
Aortic Valvular Vegetations	X		

The findings of aortic valvular vegetations concerning for non-infective endocarditis, with additional wedge-shaped renal, splenic and bilateral supratentorial and infratentorial infarcts are compatible with central embolic etiology. While EGPA itself is a rare disease, non-infectious vegetations have been documented in multiple cases. In a case report of a large non-infectious valvular vegetation associated with EGPA, they report cardiac complications in 50-62% of patients, most commonly in ANCA-negative cases [9]. According to Goodfield, cardiac involvement is not as rare as initially presumed, present in ≤ 44% of cases of EGPA [10]. However, it has been documented that cardiac involvement in GPA is a rarer manifestation, “estimated to affect only 5% of pediatric GPA and MPA cases" [11]. Furthermore, a cohort involving 517 patients diagnosed with GPA demonstrated cardiac manifestations in as little as 3.3% of patients [12]. Cardiac involvement may be subclinical or the principal cause of symptoms such as in our patient, and manifestations include but are not limited to pericarditis, arteritis, myocarditis, valvulitis and arrhythmias. Considering the high mortality of cardiac valvular involvement in patients with GPA, implementing echocardiography in standard baseline assessment may prove beneficial to prevent fatal complications.

According to the American College of Rheumatology diagnostic criteria, our patient met the criteria for GPA with findings of oral ulcers and an abnormal chest radiograph. He met four of the six criteria for EGPA with eosinophilia, lung infiltrates, paranasal sinus abnormalities, and eosinophilic infiltration on skin biopsy. While our patient’s findings are consistent with a diagnosis of GPA, the prominent eosinophilia cannot be ignored. This could also be an atypical presentation of EGPA, as oral ulcers and positive C-ANCA have been reported in this population [2,8]. Another possible explanation for the presence of diagnostic criteria for both EGPA and GPA is polyangiitis overlapping syndrome.

Polyangiitis overlapping syndrome was first described in 1986 yet little has been published on the topic [13]. The predominant characteristics of both EGPA and GPA are seen including eosinophilia, pulmonary infiltrates or cavitary lesions, chronic sinusitis and renal issues [14]. One literature review found that about 15 cases have been reported with the majority having eosinophilia along with positive C-ANCA, as seen in our patient [14]. A case report written in 2017 by Surendran et al. describes a 45-year-old female who like our patient presented with a history of polyarthralgia, respiratory symptoms, and edema. In her case, distinguishing between the vasculitides was increasingly difficult given conflicting supporting evidence including polyarthritis, pan-sinusitis, positive C-ANCA, rapidly progressive glomerulonephritis and mononeuritis multiplex. By meeting the criteria for both GPA and EGPA, she was diagnosed with polyangiitis overlapping syndrome [15]. Although previously reported, polyangiitis overlapping syndrome is not commonly recognized. Per the American College of Rheumatology, likely due to the rarity of this condition, there are no distinct diagnostic criteria [16]. This demonstrates a clear gap in care as early detection is paramount to prevent severe, irreversible organ damage and additionally helps guide therapies as treatment options differ between EGPA and GPA. EGPA patients are most often managed with corticosteroids and anti-IL 5 therapies such as mepolizumab whereas GPA is typically treated with a combination of steroids, biologics such as rituximab or cyclophosphamide and cytotoxic agents such as methotrexate. A review of the literature demonstrated that the majority of patients with EGPA and GPA overlap were treated with a combination of corticosteroids and immunosuppressive agents [8]. In our patient, he was managed with Solu-Medrol titrated to Prednisone on discharge and started on Rituximab infusion therapy. Most commonly cyclophosphamide, methotrexate and azathioprine appear to be immunosuppression agents of choice in combination with steroid therapy [8].

## Conclusions

This case report demonstrates a very rare presentation of vasculitis with a complicated clinical course. Our patient exhibited an array of symptoms with conflicting diagnostic criteria consistent with both EGPA and GPA. With the differing supportive evidence and inability to fulfill well-defined vasculitic syndromes, a more consistent diagnosis for our patient would be polyangiitis overlapping syndrome. This is defined as vasculitis with characteristics of both EGPA and GPA, with most cases having a positive C-ANCA and PR-3, as seen in our patient. As treatment options differ based on diagnosis, it is important to maintain awareness and have this rare condition as a differential to limit fatal complications.
